# Risk factors of psychosis in immigrant population: case report and literature review

**DOI:** 10.1192/j.eurpsy.2022.1629

**Published:** 2022-09-01

**Authors:** W. Kabtni, H. El Kefi, A. Baatout, C. Bencheikh, A. Oumaya

**Affiliations:** Hmpit, Psychiatry, Tunis, Tunisia

**Keywords:** Psychosis, african, Tunisia, immigrants

## Abstract

**Introduction:**

There is now compelling evidence that migrant groups in several countries have an elevated risk of developing psychotic disorders.

**Objectives:**

To identify risk factors for psychosis in immigrant population.

**Methods:**

case report and Computerised literature search of MEDLINE and PUBMED and PsycINFO databases was performed using the keywords: immigration, psychosis, schizophrenia.

**Results:**

Mrs AM is 22 years old, Ivorian, without any personal or family psychiatric history, married and mother of an 11 months old baby.

Because of the poor socio-economic conditions, she immigrated illegally to tunisia 3 months ago, accompanied by her husband, leaving her child in her native country. since then, she has been working in cleaning jobs with very low salaries and several conflicts in the workplace, which pushed AMto leave the job. One month before her admission, according to her husband, she became isolated, distrustful, she often watches herself in the mirror, refuses to take a shower, with some bizarre behaviors and persecutory words, then she became aggressive with her husband andneighbors, hence her admission.

The interview revealed a dissociative and delusional syndrome, vague and poorly systematized, with hallucinatory and intuitive mechanisms. In view of the subsequent evolution, the diagnosis of schizophrenia was retained. After stabilization under antipsychotic drugs, the patient asked to be repatriated to join her child.

**Conclusions:**

The evidence is still thin, and there is a clear need for further research to replicate and extend findings linking specific aspects of the social environment and risk of psychosis in migrant groups.

**Disclosure:**

No significant relationships.

